# An Electrolyte-Gated Graphene Field-Effect Transistor for Detection of Gadolinium(III) in Aqueous Media

**DOI:** 10.3390/bios13030363

**Published:** 2023-03-09

**Authors:** Charlène Gadroy, Rassen Boukraa, Nicolas Battaglini, Franck Le Derf, Nadine Mofaddel, Julien Vieillard, Benoît Piro

**Affiliations:** 1 Université de Rouen-Normandie, Campus d’Evreux, UMR-CNRS 6014, F-27000 Evreux, France; 2 Université Paris Cité, CNRS, ITODYS, F-75013 Paris, France

**Keywords:** gadolinium(III) detection, electrolyte-gated graphene transistor, DOTA, surface functionalization

## Abstract

In this work, an electrolyte-gated graphene field-effect transistor is developed for Gd^3+^ ion detection in water. The source and drain electrodes of the transistor are fabricated by photolithography on polyimide, while the graphene channel is obtained by inkjet-printing a graphene oxide ink subsequently electro-reduced to give reduced graphene oxide. The Gd^3+^-selective ligand DOTA is functionalized by an alkyne linker to be grafted by click chemistry on a gold electrode without losing its affinity for Gd^3+^. The synthesis route is fully described, and the ligand, the linker and the functionalized surface are characterized by electrochemical analysis and spectroscopy. The as functionalized electrode is used as gate in the graphene transistor so to modulate the source-drain current as a function of its potential, which is itself modulated by the concentration of Gd^3+^captured on the gate surface. The obtained sensor is able to quantify Gd^3+^ even in a sample containing several other potentially interfering ions such as Ni^2+^, Ca^2+^, Na^+^ and In^3+^. The quantification range is from 1 pM to 10 mM, with a sensitivity of 20 mV dec^−1^ expected for a trivalent ion. This paves the way for Gd^3+^ quantification in hospital or industrial wastewater.

## 1. Introduction

Chelated with hydrophilic ligands, Gadolinium(III) is widely used in hospitals as a contrast agent for brain MRI (magnetic resonance imaging). It is typically injected intravenously in the form of a complex before imaging, for a total of 15 tons each year worldwide. If chelated Gd^3+^ is considered safe, free Gd^3+^ is highly toxic (lethal dose of 0.34 mmol.kg^−1^ of body mass) because of its chemical similarities to Ca^2+^ (in particular, a similar ion radius), which has important roles in muscle or arteries contraction as well as in nerve transmission and, as other heavy metals, accumulates in the body. Additionally, chelated Gd^3+^ is metabolized and released later by patients in urine in the form of free Gd^3+^ ions, which then recover their toxicity. Several warnings have been given by the US Food and Drug Administration (FDA), the World Health Organization (WHO) and the European Medicines Agency (EMA) [1] in the last 15 years. It is, therefore, necessary to develop routine and costless on-site analytical devices able to perform continuous or frequent Gd^3+^ detection directly in hospital waste waters, in central water treatment plants, and in plants that synthesize the MRI Gd chelates that would like to control their releases in the environment.

Many Gadolinium MRI chelating agents are reported in the literature, as cyclic ionic ones such as Gadoterate (Gd^3+^ complexed with 1,4,7,10-tetraazacyclododecane-N,N′,N″,N‴-tetraacetic acid, DOTA) or Gadobutrol (Gd^3+^ complex with 2,2′,2″-(10-((2R,3S)-1,3,4-trihydroxybutan-2-yl)-1,4,7,10-tetraazacyclododecane-1,4,7-triyl)triacetate, DO3A-butrol) or linear ones such as Gadopentetate, and many others (it is known that cyclic ionic chelating agents are the most efficient). We may not consider these complexing agents only for Gd delivery in MRI, but, conversely, they can serve as selective capture probe for Gd^3+^ sensors. However, few Gadolinium sensors have been reported to date. Among optical sensors, Edogun et al. described a DNA-based fluorescence assay for lanthanide (among which Gd^3+^) ions, relying on the unquenching of a fluorophore upon complexation [2]. More recently, Pallares et al. described a luminescent assay based on the competitive decomplexation of linear chelate of Europium by Gadolinium(III) [3]. Among potentiometric sensors, Ganjali et al. [4] described a Gd^3+^ sensor using a Schiff base and displaying a sensitivity of ca. 20 mV.dec^−1^ and a detection limit of 3 × 10^−6^ M. Zamani et al. [5,6] also reported similar potentiometric electrodes showing a sensitivity of 20 mV.dec^−1^ and a LOD of 5 × 10^−7^ M. Very recently, Gadhari et al. [7] reported a DOTA-based potentiometric sensor for Gd^3+^ presenting the same sensitivity of 20 mV.dec^−1^ and a LOD of 7 × 10^−9^ M.

There are other ways than conventional potentiometry to detect ions, however. Among them, field-effect transistors (FETs) and more particularly electrolyte-gated FETs have emerged during the last decade. The functioning of these transistors is particular: the conventional dielectric material that usually sits between the gate electrode and the semiconducting layer is replaced by an electrolyte, solid [8] or liquid [9,10], in direct contact with the gate and the semiconductor. For a p-type (n-type) semiconductor, a negative (positive) gate bias causes an accumulation of anions (cations) at the electrolyte/semiconductor interface and the accumulation of the same number of positive (negative) charges within the semiconductor, forming a channel of positive (negative) charge carriers. These opposite charges in close vicinity generate very high interface capacitances (in the range 1–100 µF cm^−2^) that allow EG-FETs to operate at very low voltages, typically over a 0–2 V range. In these devices, conservation of charges applies so that the same amount is trapped at the gate and at the semiconductor interface. It means that the transistor can be controlled by functionalization of the electrolyte/semiconductor interface and by that of the gate/electrolyte interface, the latter being generally easier to achieve. Therefore, with a view to making a sensor, functionalization of the gate by a capture probe is a possibility [11,12], which is emphasized in this work.

Several EG-FETs based on organic semiconductors have been described for ion sensing [13,14]. One of their drawbacks is the relatively poor stability of the organic semiconductor immersed in aqueous media. For this reason, electrolyte-gated graphene FETs (EGGFETs) deserve investigation [15]. It is important to keep in mind that graphene is not a semiconductor, however; it is a semimetal for which valence and conduction bands meet at the so-called Dirac point of the Brillouin zone, which corresponds to the Charge Neutrality Point (CNP, corresponding to the minimum charge carriers’ density into the rGO) at which positive (holes) and negative (electrons) charge carriers are found in equal quantity. For this reason, when graphene is employed in field-effect transistors, there is no threshold voltage (V_th_) identifiable on the transfer curves, as for conventional FETs, but the transfer curves show an ambipolar behavior, with a p-branch (source-drain current due to holes transport) for applied gate bias leading to move the Fermi level *E_F_* below the CNP, and a n-branch (source-drain current due to electrons transport) for applied gate bias leading to move *E_F_* higher than the CNP. The energetic position of the Fermi level at equilibrium depends on the chemical state of graphene. Pristine graphene is supposed to show a minimum source/drain current $I_{D}^{min}$ at zero gate voltage (*V_G_* = 0 V). However, defects induced by the synthesis protocol, for instance, induce some structural defects or intercalation of doping species able to significantly shift the value of *V_G_* at which $I_{D}^{min}$ is observed [16].

EGGFETS are particularly relevant for sensing applications because of the robustness of graphene in aqueous environments compared to organic semiconductors, but mostly because of its sensitivity to electrostatic interactions, due to the ideally 2D structure (i.e., a very high surface-to-volume ratio). Thus, ion sensors based on EGGFETs have been reported. For example, Maehashi et al. [17] described in 2013 an ionophore-modified EGGFET for selective K^+^ detection, demonstrating a left-shift (toward lesser positive potentials) of the CNP with a sensitivity of ca. −8 mV.dec^−1^ between 10 nM and 1.0 mM. Very recently, Wang et al. [18] reported an aptamer-based EGGFET for Cu^2+^ detection presenting a detection range from 10 nM to 3 μM and a sensitivity of the CNP position estimated at ca. −30 mV.dec^−1^.

These latter two EGGFETs were fabricated by conventional methods, i.e., subtractive photolithography in a clean room. For the sake of cost saving and simplicity, in view of applications, it is today more than ever pertinent to develop energy-saving additive manufacturing processes for these devices, such as inkjet printing. However, pristine graphene is extremely tricky to print directly as it is, because it cannot be processed in solution (or even stable suspension) without the use of surfactants or other additives that adsorb on the graphene flakes and modify their electrical properties. For this reason, the use of graphene oxide (GO) is preferred, which can form stable suspensions in water without any additive [19]. Due to GO being poorly conductive, it is necessary to retrieve part of the electrical conductivity of graphene by reduction from GO to rGO (reduced graphene oxide) after printing. We showed in a previous work that the electrochemical reduction of GO leads to notable change in the position of the CNP, which can be shifted in a controlled manner over a range of 1 V from its initial position. This makes it possible to finely tune the doping level of rGO as a function of the Coulomb charge passed, and also makes it possible to modulate the mobility of charge carriers over more than one order of magnitude [20].

**Scheme 1:**
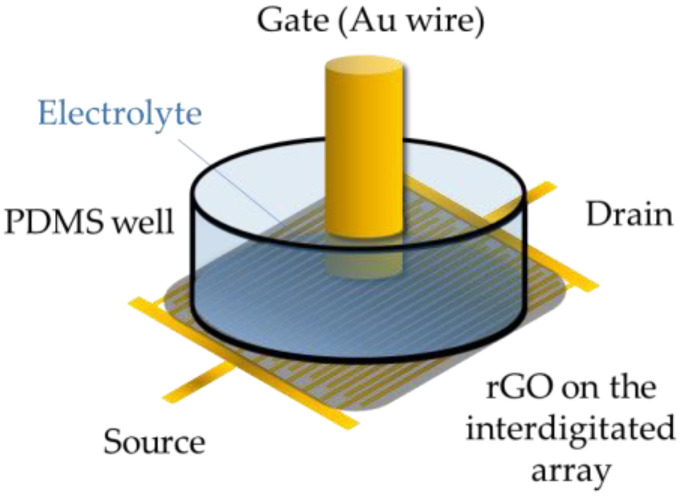
Illustration of the setup: the EGGFET device consists of an interdigitated source-drain layout with a total channel width of 30 mm and a length (distance between fingers) of 10 μm). GO was printed on top, and then electroreduced to rGO. For measurements, a PDMS well was sealed on top of the interdigitated array and filled with the electrolyte, and a gold wire (∅ = 1 mm) was dipped into the electrolyte used as gate.

In this work, an EGGFET fabricated on polyimide (Kapton^®^_,_ DuPont, Wilmington, DE, USA) wafer was used instead of a conventional silicon wafer, onto which GO was inkjet-printed on top and in between the source-drain interdigitated contacts, then electroreduced into rGO to form the conductive channel (Scheme 1). This device was applied for the detection and quantification of free Gd^3+^. For the sake of simplicity, we used a gold wire as gate, functionalized by a DOTA derivative and dipped into the aqueous electrolyte that covers the channel of the transistor. After estimation of the association constant of free Gd^3+^, the resulting transistor was electrically characterized in 0.1 M KCl, then changes in its transfer curves were recorded for various concentrations of added Gd^3+^.

## 2. Materials and Methods

### 2.1. Instruments and Procedures

Chemicals, reagents, synthesis and routine characterization procedures are detailed in Appendix A, Appendix B and Appendix C.

Isothermal Titration Calorimetry (ITC) was performed using a high-sensitivity MicroCal PEAQ-ITC Malvern-Panalytical apparatus. Several solutions were prepared before-hand: 50 µM DOTA, 50 µM DO3A-alkyne (Product *7*, see Appendix A, Appendix B and Appendix C), 500 µM GdCl_3_, 6H_2_O, 50 µM CaCl_2_. The DOTA or DO3A-alkyne solutions were inserted into the calorimeter and the system was let to equilibrate for 5 min. The measurements were started by one injection of 0.4 µL of Gd^3+^ (or Ca^2+^) followed by 18 injections of 2 µL of the same ion, every 3 min. The electrochemical characterizations of the adsorption in the gold threads were made by Cyclic Voltammetry (CV) on an Autolab PGSTAT30 potentiostat using an equimolar solution of K_4_FeCN_6_/K_3_FeCN_6_ (10^−2^ M) in 0.1 M KCl. Experiments were performed using Au wires as working electrode (∅ = 1 mm), a lab-made Ag/AgCl reference electrode and a stainless-steel grid as counter electrode.

The transistor electrodes were patterned from a one-step photolithographic process followed by e-beam-assisted evaporation of 100 nm gold layer directly on the polyimide substrate, to define an interdigitated source-drain layout with a channel width/length (W/L) ratio of 3000 (W = 30,000 μm, L = 10 μm). The ink was formulated from a commercial GO powder (from Nanografi Co. Inc, Ankara, Turkey) suspended under sonication into a mix of 50% (*v*/*v*) DI-water, 30%(*v*/*v*) 1-propanol and 20% (*v*/*v*) ethylene glycol. It was printed on the interdigitated source and drain electrodes using a Dimatix inkjet printer 2850 (DMP 2850). Three layers were subsequently printed to form a homogeneous GO channel.

The reduction of printed GO to reduced (rGO) was performed on a Biologic SP-240 potentiostat by chronocoulometry using a Ag/AgCl reference electrode, a stainless-steel grid as counter electrode and the short-circuited source and drain contacts as working electrode. A controlled constant potential of −1.3 V was applied up to the appropriate reduction charge (0.5 mC). Transistor characterizations were performed with a Keithley 4200 A semiconductor characterization system, and the transfer curves with 0.1 M KCl or PBS as electrolyte were acquired for various concentrations of Gd^3+^ and/or other supposed interfering ions.

### 2.2. Protocol for DO3A-Alkyne Immobilization

Au electrodes were cleaned with acetone and isopropanol then shortly passed under an H_2_ flame to remove all organic impurities. Some electrodes were coated with an insulating varnish then polished on diamond polishing cloths at one end to leave only an Au disk of the diameter of the wire. 1-azido-6-thiohexane (Product 3) was dissolved in 1 mL MilliQ water + a minimum (50 µL) of acetonitrile and the Au wires were dipped into this solution for 24 h, then thoroughly washed with ultrapure water under sonication and dried under argon before being stored in a 2 mL Eppendorf under argon atmosphere. For coupling of the modified DO3A-alkyne (Product 7, see Appendix A, Appendix B and Appendix C), an Eppendorf was filled with sodium ascorbate (20 mg, 0.101 mmol) in 1 mL degassed MilliQ water. In another Eppendorf, copper(II) sulphate pentahydrate (0.005 g, 0.02 mmol) was dissolved in 1 mL degassed MilliQ water. DO3A-alkyne was then dissolved in 250 µL of the sodium ascorbate solution +25 µL of the copper(II) sulphate pentahydrate solution and the Au wire was put in the Eppendorf and left for 24 h. After this step, Au electrodes were washed with ultrapure water and dried under argon before being stored back in an Argon-flushed Eppendorf.

## 3. Results and Discussion

### 3.1. Determination of the Affinity Constants between Gd^3+^ and DOTA or DO3A-Alkyne

Au electrodes were cleaned with acetone and isopropanol and then shortly passed under an H_2_ flame to remove all organic impurities. Some electrodes were coated with an insulating varnish. Before using the DO3A-alkyne chelator in the EGGFET sensor, we estimated by microcalorimetry the formation constants of DOTA and DO3A-alkyne complexes with Gd^3+^ and Ca^2+^, the latter being, as explained in the introduction, a known competitor of Gd^3+^. The heat variations were plotted as a function of the [DOTA]/[Gd^3+^], [DOTA]/[Ca^2+^] and [DO3A-alkyne]/[Gd^3+^] molar ratios, from which the formation constants K_f_ were estimated by adjusting the isotherms. For complexation of Ca^2+^ by DOTA, the heat changes were extremely low, indicating a very low affinity; therefore, the formation constant K_f(DOTA/Ca2+)_ was not quantified.

**Figure 1:**
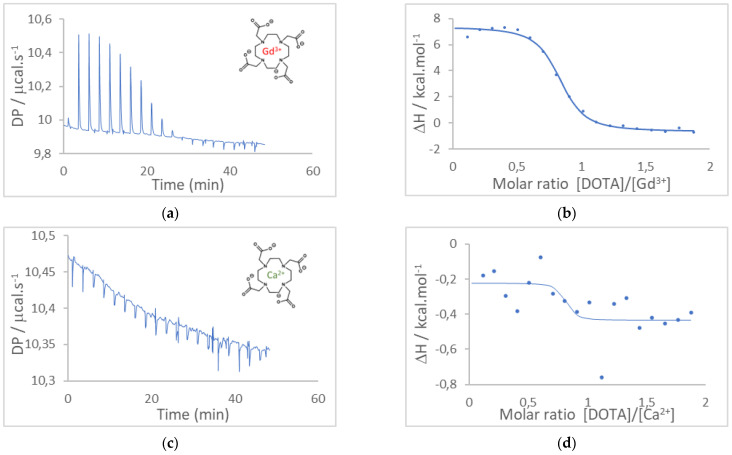

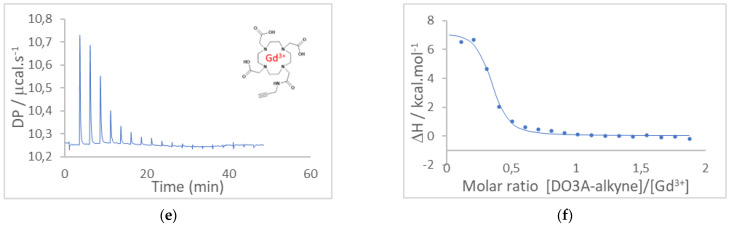
Isothermal titration calorimetry (ITC) results. (**a**) Raw ITC results for DOTA/Gd^3+^ complexation and (**b**) corresponding enthalpy fit. (**c**) Raw ITC results for DOTA/Ca^2+^ and (**d**) corresponding enthalpy fit. (**e**) Raw ITC results for DO3A-alkyne/Gd^3+^ and (**f**) corresponding enthalpy fit. Solutions were prepared before-hand: 50 µM DOTA or 50 µM DO3A-alkyne, 500 µM GdCl_3_, 50 µM CaCl_2_. The DOTA or DO3A-alkyne solutions were inserted into the calorimeter and the system was let to equilibrate.

Conversely, for complexation of Gd^3+^ by DOTA and DO3A-alkyne, heat changes were both very significant and made it possible to estimate the respective formation constants K_f(DOTA/Gd3+)_ and K_f(DO3A-alkyne/Gd3+)_. In our experimental conditions, T = 25 °C, MilliQ water (pH 5.5) and [DOTA] = 50 × 10^−6^ M, K_f(DOTA/Gd3+)_ was estimated at (2.5 ± 0.6) × 10^6^ and K_f(DO3A/Gd3+)_ was estimated at (1.4 ± 0.3) × 10^6^. These values are consistent with the literature and demonstrate the high affinity of DOTA and DO3A-alkyne for Gd^3+^; these constants are expected significantly greater in higher ionic strength media [21,22,23,24]. Therefore, DO3A-alkyne is suitable for surface modification and for Gd^3+^ detection. The microcalorimetry results and the corresponding adjustments are given in Figure 1.

### 3.2. Functionalization of the Gate Electrode

**Figure 2:**
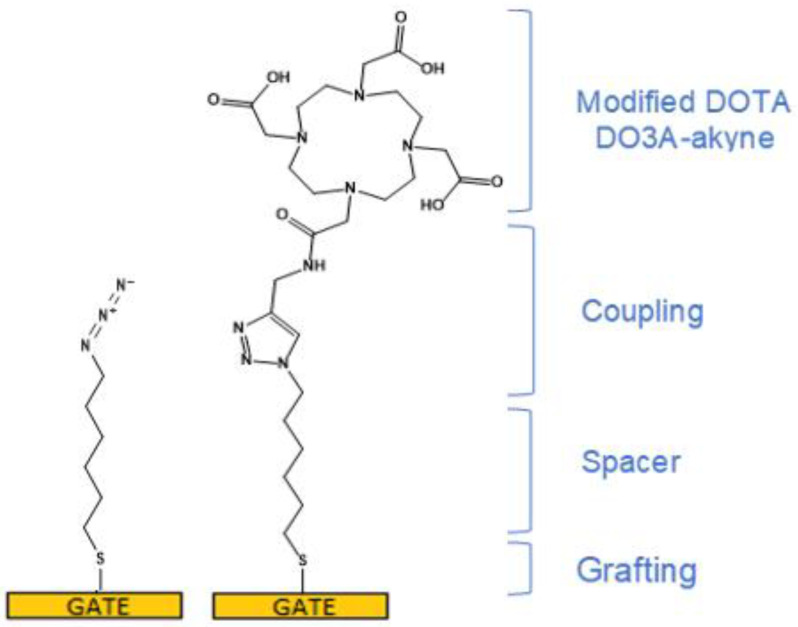
Immobilization scheme of DO3A-alkyne on a Gold gate surface. First, a C_6_ alkylthiol (1-azido-6-thiohexane) was immobilized on the Au surface (**left**) then coupled with Product 7 (DO3A-alkyne) through the copper-catalyzed azide-alkyne cycloaddition (**right**) [26].

**Figure A1:**
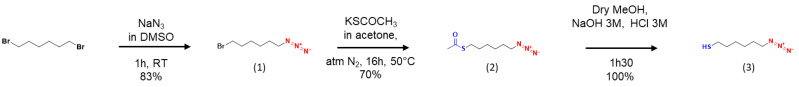
Synthesis of the 1-azido-6-thiohexane.

**Figure A6:**
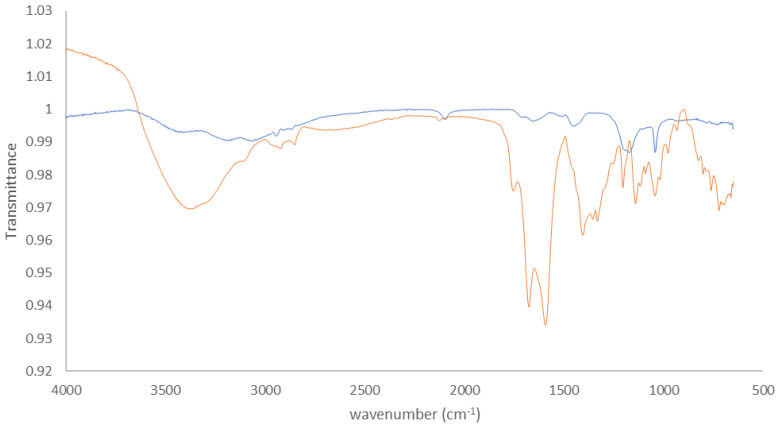
FTIR spectra of the Au surface functionalized with 1-azido-6-thiohexane (blue) and with DO3A-alkyne (orange). The FTIR spectra from Au is subtracted from modified surface.

**Figure A7:**
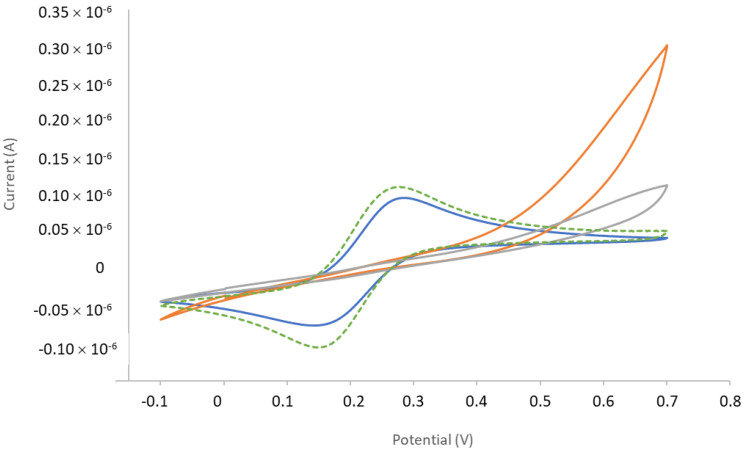
Cyclic voltammograms (CVs) of the ferri/ferrocyanide couple on a bare Au electrode (dotted green line), functionalized with 1-azido-6-thiohexane (orange line) and after coupling of DO3A-alkyne on the 1-azido-6-thiohexane-functionalized electrode (grey line). The blue CV corresponds to a bare Au electrode let to react on DO3A-alkyne under the same conditions as the 1-azido-6-thiohexane-functionalized electrode. Electrolyte: 0.1 M KCl; 0.01 M Fe(CN)_6_^3−^/Fe(CN)_6_^4−^ as potassium salt. Au wire electrodes (∅ = 1 mm).

Gate functionalization was performed as described in Section 2 and illustrated in Figure 2. XPS, FTIR and cyclic voltammetry techniques were used to characterize the grafting and coupling steps. XPS and FTIR experiments were carried out on commercial planar Au electrodes (sputtered gold on glass plates). Data analyses are detailed in Appendix C. In summary, complete chemisorption of the spacer (1-azido-6-thiohexane) and the ligand (DO3A-alkyne) on Au are confirmed by XPS analysis (Figure A1). Secondly, the atomic composition of the electrode surface after the click coupling was consistent with the addition of DO3A-alkyne to the electrode. This indicates that the surface coupling conditions were sufficiently mild to proceed at the solid-liquid interface [25]. Additionally, the N_1s_ XPS spectrum unambiguously confirmed the attachment of DO3A-alkyne to the spacer. However, FTIR spectra of the DO3A-alkyne-functionalized surface (Figure A6) showed that most but not all azido groups reacted upon coupling with the DO3A-alkyne. Cyclic voltammetry (Appendix C, Figure A7) showed that simple adsorption of DO3A in these conditions does not lead to any electrochemical blocking of the interface. Conversely, grafting of the 1-azido-6-thiohexane leads to a partial blocking; when followed by coupling with DO3A-alkyne, the blocking is even more pronounced. These results show the efficiency of the 1-azido-6-thiohexane grafting and that of the DO3A-alkyne coupling, under the form of a dense monolayer, so that the surface density of DO3A could be estimated in the range 5 × 10^−11^–1 × 10^−10^ mol.cm^−2^ (values based on spatial calculations).

### 3.3. Gd^3+^ Sensing Using the EGGFET

**Figure 3:**
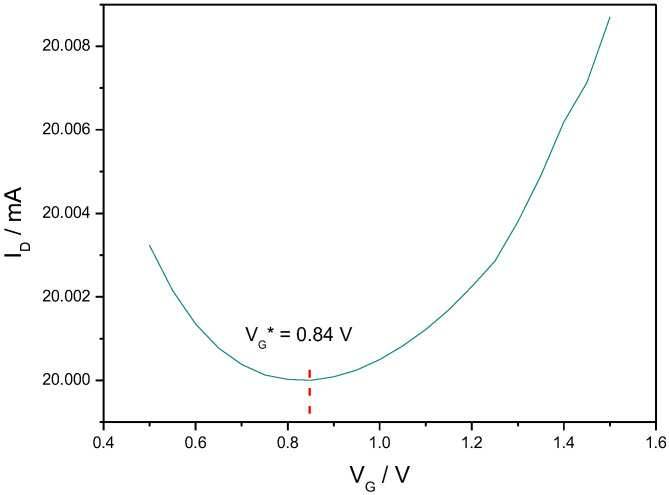
Typical transfer characteristic recorded for V_D_ = 0.9 V on an inkjet-printed rGO EGGFET, with a gate diameter of 1 mm (area of 7.85 × 10^−3^ cm^2^) and an rGO area of 5 × 10^−3^ cm^2^. V_G_* = 0.84 V. Electrolyte: PBS. T = 25 °C.

We previously described the electrical behavior of EGGFETs [20]. The transfer characteristics of such transistors is expected to consist of a U-shape curve with a p-branch on the left-hand side corresponding to the contribution of holes to conductivity, and a n-branch on the right-hand side corresponding to the contribution of electrons to conductivity. The minimum of the source/drain current $I_{D}^{min}$ is obtained for a specific value of the gate voltage V_G_* which relies on the doping state of rGO at equilibrium. For a p-doped material (i.e., *E_F_* below CNP), the value of V_G_* is expected to be found at positive gate voltage, as shown on Figure 3). For a 1 mm gate wire (area of 7.85 × 10^−3^ cm^2^, while the rGO area is 5 × 10^−3^ cm^2^), we found V_G_* = 0.84 V. For smaller gates (0.5 and 0.25 mm in diameter, i.e., 1.96 × 10^−3^ cm^2^ and 0.49 × 10^−3^ cm^2^, respectively), V_G_*was shifted to lower voltages but, as expected from the conservation of charges between the gate/electrolyte and the electrolyte/rGO interface, the gating effect was lower as well as the drain current (results not shown). For this reason, all experiments were performed with 1 mm gates. Under these conditions, the amplitude of the drain current changes was of about 10 μA upon a gate voltage amplitude of 1 V. It must be noted that the output curves are very stable with time: after two runs of gate voltage sweep, the transfer characteristic is stable over several hours of experiment.

As for organic electrolyte-gated transistors [27], the potential difference between the source and the gate electrode is the sum of the potential drop at the gate/electrolyte and at the electrolyte/graphene interfaces. Therefore, if we assume that nothing is changed at the graphene interface, any change at the gate results in a change in the gate voltage, i.e., in a translation of the transfer characteristic along the gate voltage axis [20]. It has been shown [13] that, for ion detection on electrolyte-gated or electrochemical FETs, the gate shift is proportional to the logarithm of the ion concentration as expected with the Nernst law. This behavior can be directly translated into a V_G_* change in our case:

V_G_* = *a* log [Gd^3+^] + V_G_*^0^, (1)

**Figure 4:**
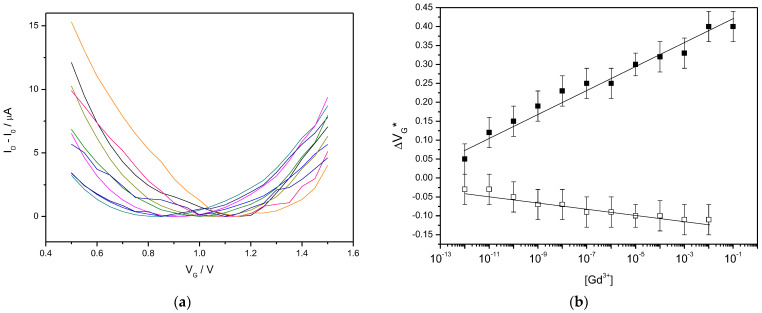
(**a**) Transfer curves of a DO3A-alkyne-functionalized EGGFET, for *V_G_* swept from +1.5 V down to +0.5 V, for Gd^3+^ concentrations ranging from 10^−12^ to 10^−3^ M, from left to right (on color per concentration). The minimum current $I_{D}^{min}$ as subtracted from all curves so that all minima are artificially set to 0. (**b**) Change of the position of the minimum V_G_* of the above transfer curves for various Gd^3+^ concentrations, on a DO3A-alkyne-functionalized EGGFET (black squares) and on a non-functionalized EGGFET (white squares). The electrolyte always contains 0.1 M KCl.

Δ V_G_*= V_G_* − V_G_*^0^ = *a* log [Gd^3+^], (2)

with *a,* the proportionality constant (assimilated to a sensitivity) and V_G_*^0^ the position of the minimum of the transfer curve on the *V_G_* axis in the absence of Gd^3+^ in the electrolyte. Figure 4a shows the series of transfer curves obtained for increasing Gd^3+^ concentrations while Figure 4b shows the evolution of V_G_* as a function of these concentrations. We found a value of *a* = 29 mv dec^−1^ for the shift of V_G_* in these conditions. The Figure 4c shows a blank experiment obtained under the same conditions as for Figure 4b but using a bare gate electrode instead of a DO3A-alkyne-functionalized one. In these conditions, the shift of V_G_* was found poorly dependent on the Ga^3+^ concentration, with a slope of ca. −8 mV.dec^−1^. Note that, in Figure 4a, the drain current $I_{D}^{min}$ was subtracted from the measured drain current *I_D_* for all *V_D_* to make easier the comparison between each minimum. Note that, to some extent, the position of minimum of the transfer curve moves with the value of the drain voltage. For the sake of comparison, we took care to set a constant value of *V_D_* for all our measurements.

**Figure 5:**
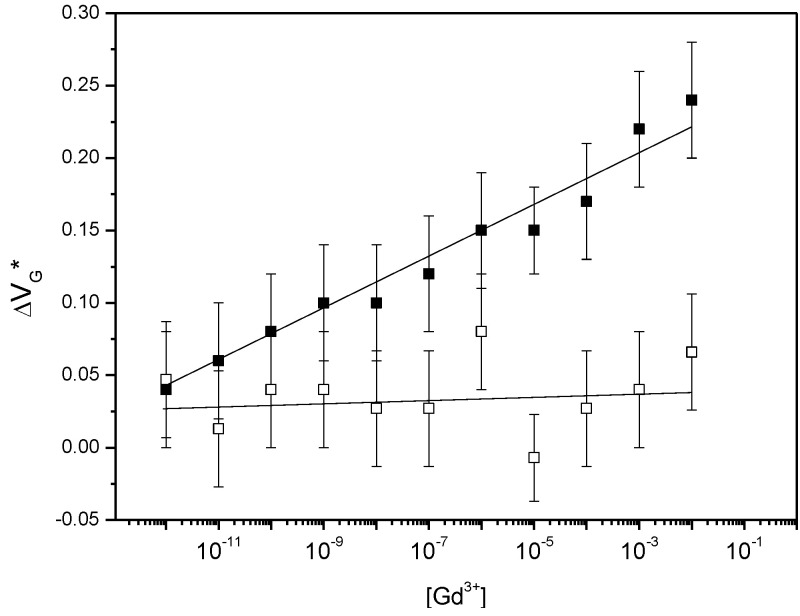
Position of the minimum of the transfer curve for various Gd^3+^ or Ca^2+^ concentrations, on a DO3A-alkyne functionalized EGGFET in an electrolyte containing Ni^2+^, Ca^2+^, Na^+^ and In^3+^ ions at 10^−6^ M each + 0.1 M KCl (black squares) and for various Ca^2+^ (white squares) in an electrolyte containing 0.1 M KCl.

Another experiment was performed on a DO3A-alkyne-functionalized EGGFET for which the concentration of interfering ion Ca^2+^ was changed, and on a DO3A-functionalized EGGFET for which the concentration of Gd^3+^ was changed in a medium containing all the potential interfering ions Ni^2+^, Ca^2+^, Na^+^ et In^3+^ at 10^−6^ M, plus 0.1 M KCl (Figure 5). These experiments show a V_G_* that is insensitive to the concentration of Ca^2+^ ions, whereas it shifts of ca. 20 mV.dec^−1^ for Gd^3+^ in a mixture of other ions.

As shown, even if precisely determined, and considering the standard deviation, the limit of detection of our DO3A-functionalized EGGFET is below 10^−12^ M and the limit of quantification sits between 10^−12^ and 10^−11^ M. The dynamic range is very large, from 10^−12^ to 10^−2^ M, and the sensor is selective to Gd^3+^. The average concentration of Gd(III) in effluents of a hospital offering MRI and radiotherapies sits between 10 and 20 μg·L^−1^, corresponding to 60–120 nM.

We did not have the opportunity to work on real samples such as hospital wastewater, for regulatory reasons. However, the results given here show that the approach consisting of functionalizing an EGGFET by DOTA or DOTA derivatives, which was yet not reported, is promising.

## 4. Conclusions

We developed here a polyimide-supported EGGFET in which the channel is made of reduced graphene oxide, obtained by electro-reduction of an inkjet-printed lab-made graphene oxide ink. The gold gate electrode was functionalized by a DOTA-like ligand, known to be specific to Gd^3+^ ion. The synthesis of the DOTA-alkyne ligand and of the azido-functionalized alkylthiol spacer are described. The ligand is grafted on the Au gate of the transistor through a spacer so to guarantee a sufficient accessibility of the DOTA to Gd^3+^ while keeping it sufficiently close to the electrode in order to efficiently influence its potential. The constants of formation of the DOTA-Gd^3+^ complex and of its modified form DO3A-alkyne-Gd^3+^ were evaluated using isothermal titration calorimetry (ITC), showing *K_f_* values in the order of 10^6^ in our operating conditions, values that make them pertinent for being used as efficient ligands in the developed EGGFETs. Their electrical characteristics were measured with or without Gd^3+^, with or without DO3A-alkyne, and with or without potential interfering ions. The results show that the device is poorly sensitive to Gd^3+^ if a non-functionalized bare Au gate is used, whereas it is sensitive to Gd^3+^ using a DO3A-alkyne-modified gate, even in a mixture of potentially interfering monovalent, bivalent or trivalent ions of comparable size, with a shift of the transfer curve along the gate voltage axis of 20 mV.dec^−1^ in these conditions. These results pave the way for using DOTA- or DO3A-alkyne-modified EGGFETs for quality control of hospital or industry wastewaters. The next step in the development will be the amplification of the gate voltage shift into a drain current change, at constant *V_D_* and *V_G_*, and application on real hospitals wastewaters. The selectivity against Pb^2+^, an ion that can also be found in hospital wastewaters and for which DOTA-like ligands were shown to present good affinity [24], will be also investigated.

## Figures and Tables

**Figure A2:**
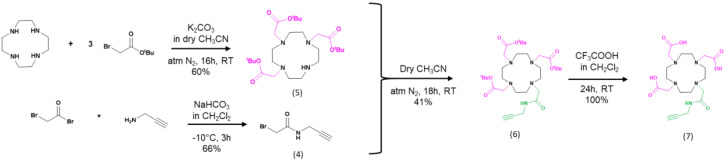
Synthesis of modified DOTA to immobilize DOTA on transistor.

**Figure A3:**
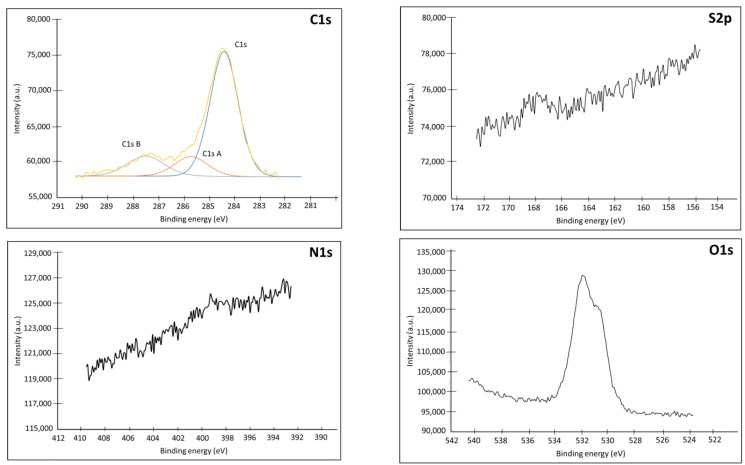
XPS spectra (C1s, S2p, N1s, O1s) of a bare Au plate electrode.

**Figure A4:**
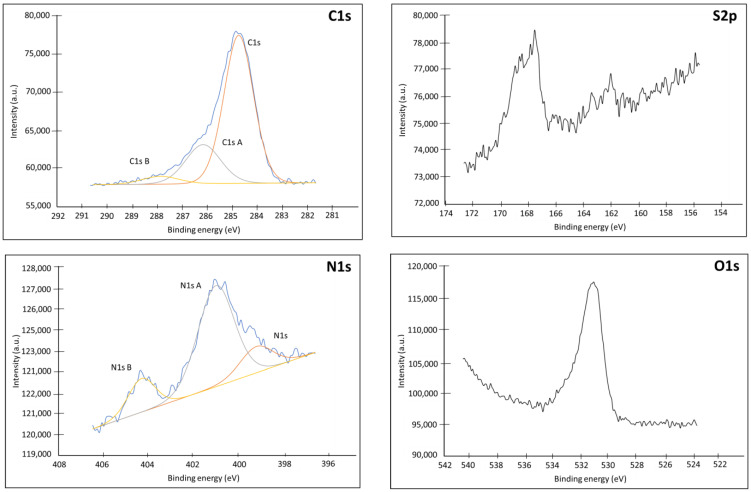
XPS spectra (C1s, S2p, N1s, O1s) of an Au plate electrode modified with 1-azido-6-thiohexane (Product 3).

**Figure A5:**
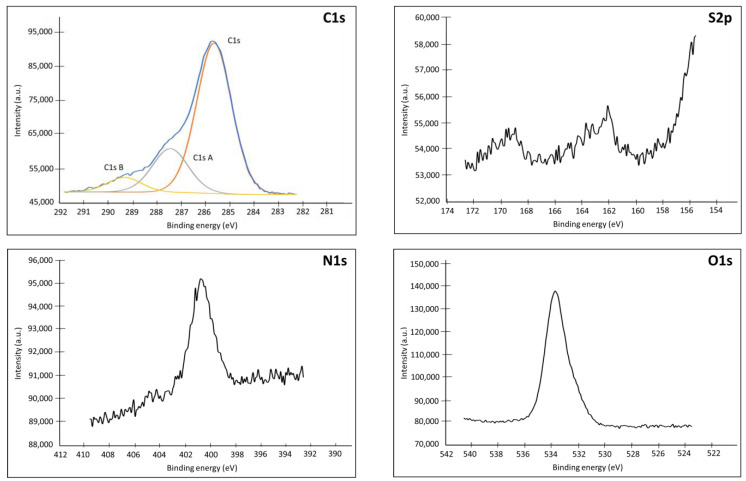
XPS spectra (C1s, S2p, N1s, O1s) of an Au plate electrode modified with Product 7.

## Data Availability

The data presented in this study are available within the article and Appendix A, Appendix B and Appendix C.

## Appendix A. Chemicals, Reagents and Routine Procedures

All chemicals were used as received without any further purification. Gold wires (99.95%, 1 mm, 0.5 mm and 0.25 in diameter) were purchased from Sigma-Aldrich. 1,6-dibromohexane (98%), acetonitrile dry (99.9% extra dry over molecular sieve), methanol dry (99.9% extra dry over molecular sieve) and n-hexane (95%) were purchased from Acros Organics. Extra-dry acetonitrile and methanol were used with syringe and needle under dry inert atmosphere only. Potassium thioacetate (98%), gadolinium(III) chloride hexahydrate (99%), 1-benzylimidazole (99%), sodium azide (99.5%), hydrochloric acid (37%), sodium hydroxide (98%), bromohydric acid (48%), 1,4,7,10-tetraazacyclododecane (cyclen) (97%) and magnesium sulphate (99.5%) were purchased from Sigma-Aldrich. Calcium (II) chloride dihydrate (99.6%) was purchased from AnalaR NORMAPUR. Acetone HPLC-grade (99.5%), acetonitrile HPLC-grade (99.9%) and dichloromethane HPLC-grade (99.8%) were purchased from Fisher. Diethyl ether (99.7%), methanol HPLC-grade (99.8%,) were purchased from VWR Chemicals. Fresh Phosphate buffer saline (PBS) was prepared daily from tablets (Sigma Aldrich) dissolved in sterile MilliQ water (1 tablet in 200 mL).

Column chromatography purifications were performed in a glass column filled with silica gel (35–70 μm) from Acros Organic. The spots were stained with vanillin and visualized under a UV lamp (λ = 254 nm). High Resolution Mass Spectrometry (HRMS) were made by a Thermo LTQ Orbitrap XL apparatus equipped with an ESI source for all finals and large molecules or on a LCT Premier XE benchtop orthogonal acceleration time-of-flight mass spectrometer (oa-TOFMS, Waters Micromass) for all small molecules. This equipment has an electrospray source and a positive/negative mode. ^1^H and ^13^C NMR spectra were recorded with an Ultrashield 300 MHz Bruker NMR spectrometer. All chemical shifts are expressed in parts per million (ppm) and the calibration was made by the residual solvent. All spectra for ^1^H NMR were measured between 0 and 16 ppm, with 16 scans and 2 s of relaxation time. All spectra for ^13^C NMR were measured between 0 and 250 ppm, with 30,000 scans and 5 s of relaxation time. The following names were used as abbreviations in the spectral description: singlet (s), doublet (d), triplet (t), doublet of doublet (dd), multiplet (m). Changes in the functional group after nucleophile substitution were identified by an Attenuated Total Reflectance tool with Fourier Transform Infra-Red Spectroscopy (ATR-FTIR) analysis using a ThermoFischer Nicolet iZ10 FTIR spectrometer. All spectra were measured between 4000 cm^−1^ and 650 cm^−1^; backgrounds were measured in air and spectra treated with OMNIC software. Adsorption of molecules on gold were characterized by ATR-FTIR using a Nicolet 8700 FTIR spectrometer with a Varig ATR (ATR mono-reflection with variable angle of incidence) dedicated to monolayers. X-ray photoelectron spectroscopy (XPS) measurements were made using a Thermo ESCALAB spectrometer using a monochromic Al Kα source at 1486.6 eV.

## Appendix B. Synthesis Protocols

The following molecules were synthesized as previously described [28,29,30,31,32]. The routes to obtain products 1, 2 and 3 are illustrated in Figure A1, and the routes to obtain products 4, 5, 6 and 7 are illustrated in Figure A2.

*Synthesis of 1-azido-6-bromohexane* (Product 1). To a solution of 1,6-dibromohexane (12.92 g, 52.94 mmol, 2.5 eq.) was added a solution of sodium azide (1.3175 g, 20.26 mmol, 1 eq.) dissolved in 30 mL DMSO. The solution was stirred 1 h at room temperature and stopped with 100 mL of ultrapure water. A liquid–liquid extraction was performed with diethyl ether (3 × 100 mL). The organic phase was recovered and dried with magnesium sulphate, and then filtered on filter paper. The product was purified by chromatography column with a mixture of hexane/dichloromethane and then concentrated to afford the product (1) as a light-yellow oil (3.4825 g, 83.4%). **IR (ν, cm^−1^)**: 2937, 2860, 2094, 1455, 1348, 1245,728. **^1^H NMR (300 MHz, CDCl_3_)**: δ 3.40 (t, 2H), 3.26 (t, 2H), 1.85 (t, 2H), 1.59 (t, 2H), 1.42 (m, 4H). **^13^C NMR (90 MHz, CDCl_3_)**: δ 51.29, 33.71, 32.55, 28.69, 27.68, 25.89.

*Synthesis of the 1-azido-6-acetylthiohexane* (Product 2). To the product (1) (1.00 g, 4.85 mmol, 1 eq.) was added a solution of potassium thioacetate (1.6617 g, 14.56 mmol, 3 eq.) dissolved in 10 mL HPLC grade acetone. The mixture was stirred and heated at 50 °C for 16 h and then concentrated in vacuum. The product was dissolved in 100 mL dichloromethane, and a liquid–liquid extraction was performed with ultrapure water (3 × 100 mL). The organic phase was recovered and dried with magnesium sulphate, and then filtered on filter paper. The product was purified by chromatographic column with a mixture of hexane/dichloromethane and then concentrated to afford the product (2) as a yellow oil. (0.6772 g, 70%). **IR (ν, cm^−1^)**: 2923, 2860, 2094, 1710, 1427, 1362, 1118, 785. **^1^H NMR (300 MHz, CDCl_3_)**: δ 3.21 (t, 2H), 2.81 (t, 2H), 2.27 (s, 3H), 1.53 (m, 4H), 1.34 (m, 4H). **^13^C NMR (90 MHz, CDCl_3_)**: δ 195.80, 51.27, 30.59, 29.36, 28.88, 28.64, 28.21, 26.18.

*Synthesis of the 1-azido-6-thiohexane* (Product 3). The product (2) (12.8 mg, 0.063 mmol, 1 eq.) was dissolved in 5 mL dry methanol under inert atmosphere. This mixture was stirred at room temperature. A solution of sodium hydroxide was added (3 M, 10 mL), and then, the mixture was stirred at room temperature for 1.5 h. Then, a solution of hydrochloric acid (3 M, 20 mL) was added, and stirred at room temperature for 1.5 h. A liquid–liquid extraction was performed with dichloromethane (3 × 50 mL), the organic phase was recovered and dried with magnesium sulphate, and then filtered on filter paper to afford the product (3) as a colorless oil (10.06 mg, 99.3%). **IR (ν, cm^−1^)**: 3000, 2922, 2852, 2376, 2095, 1464, 1260, 1016, 800. **^1^H NMR (300 MHz, CDCl_3_)**: δ 3.27 (t, 2H), 2.68 (t, 2H), 1.70 (t, 2H), 1.61 (t, 2H), 1.50 (s, 1H), 1.40 (m, 4H). **^13^C NMR (90 MHz, CDCl_3_)**: δ 51.31, 37.26, 32.09, 28.62, 27.04, 26.28.

*Synthesis of 2-bromo-N-(prop-2-ynyl)acetamide* (Product 4).

Propargylamine (1.16 mL, 18.16 mmol, 1 eq.) was dissolved in 8 mL dichloromethane, then cooled to −10 °C. A solution of saturated aqueous sodium bicarbonate (NaHCO_3_) (8 mL) was added under vigorous stirring. Then, bromoacetyl bromide (1.9 mL, 21.79 mmol, 1.2 eq.) was slowly added and the reaction mixture was slowly brought back to room temperature. After 3 h, the two phases were separated, the organic phase was dried by sodium sulphate and the solvent was removed under vacuum to afford the pure product (4) as a brown solid (2.1 g, 66%). **^1^H NMR (300 MHz, CDCl_3_)**: δ 6.72 (s, 1H, NH), 4.08 (dd, 2H), 3.90 (s, 2H, H1), 2.28 (t, 1H). **^13^C NMR (75 MHz, CDCl_3_)**: δ 165.1, 78.5, 72.2, 29.9, 28.6. **TOF HRMS (ESI Positive)**: *m/z* Calc. for C_5_H_6_BrNONa [M + Na]^+^: 197.9525, Found 198.0366.

*Synthesis of tri-tert-butyl 2,2′,2″-(1,4,7,10-tetraazacyclododecane-1,4,7-triyl)triacetate* (Product 5). 1,4,7,10-tetraazacyclododecane (0.5 g, 2.9 mmol, 1 eq.) and potassium carbonate (1.2024 g, 8.7 mmol, 3 eq.) were dissolved in 13 mL dry acetonitrile under nitrogen atmosphere. Then, a solution of tert-butylbromoacetate (1.275 mL, 8.7 mmol, 3 eq.) was added slowly and the mixture was stirred at room temperature for 16 h. The precipitate was filtered and washed by dichloromethane. The solvent was evaporated under reduced pressure and the resulting solid was purified by column chromatography (CH_2_Cl_2_/MeOH, 90:10, *v*/*v*). The resulting compound (5) was a white solid (900 mg, 60%). **^1^H NMR (300 MHz, CDCl_3_):** δ 10.04 (s, 1H, NH), 3.52–3.35 (m, 4H), 3.30 (s, 2H), 3.09 (s, 4H), 2.91–2.88 (s, 12H), 1.45 (s, 27H). **^13^C NMR (75 MHz, CDCl_3_):** δ 170.9, 170.0, 82.2, 81.9, 78.0, 65.9, 58.6, 51.7, 49.6, 47.9, 30.1, 28.6. **TOF HRMS (ESI Positive):** *m/z* Calc. for C_26_H_51_N_4_O_6_ [M + H] +: 515.3809, Found 515.3807.

Synthesis of tri-tert-butyl 2,2′,2″-(10-(2-oxo-2(prop-2-yn-1-ylamino)ethyl)-1,4,7,10-tetraazacyclododecane-1,4,7-triyl)triacetate (Product 6). Product (4) (0.5 g, 0.9714 mmol, 1 eq.), product (5) (0.171 g, 0.9714 mmol, 1 eq.) and K_2_CO_3_ (0.1743 g, 0.9714 mmol, 1eq.) were all dissolved in 10 mL dry acetonitrile under N_2_ atmosphere, and the mixture was stirred for 18 h at room temperature. The solvent was reduced under vacuum. The residual solid was dissolved in chloroform and washed 3 times by a solution of K_2_CO_3_ 5%, and then by a solution of NaOH (1 M, 60 mL). The organic phase was dried and filtered then the solvent was reduced under vacuum. The residue was purified by column chromatography (CH_2_Cl_2_/MeOH, 90:10, *v*/*v*). The product (6) was obtained as a white solid (0.330 g, 93%). **^1^H NMR (300 MHz, CDCl_3_)**: δ 8.91 (t, J = 5.4 Hz, 1H, NH), 3.94 (s, 2H), 3.47 (s, 5H), 3.09–1.86 (m, 20H), 1.45 (s, 27H). **^13^C NMR (75 MHz, CDCl_3_)**: δ 171.7, 171.4, 81.3, 81.0, 80.9, 70.3, 57.9, 56.7, 56.4, 55.4, 53.9, 52.6, 52.3, 28.7, 28.4, 28.4. **MS (ESI Positive)**: *m/z* 610.407 [M + H] ^+^.

Synthesis of 2,2′,2″-(10-(2-oxo-2(prop-2-yn-1-ylamino)ethyl)-1,4,7,10-tetraazacyclododecane-1,4,7-triyl)triacetic acid (Product 7, DO3A-alkyne). To the product (6) (0.2414 g, 0.395 mmol, 1 eq.) was added trifluoroacetic acid (TFA) (2.5 mL, 0.395 mmol, 1eq.) and the mixture was dissolved in 2.5 mL dichloromethane for 24 h at room temperature, under argon atmosphere. The solvent was removed under reduced pressure. The residue was redissolved in dichloromethane and the solvent evaporated under reduced pressure until a solid product is obtained. The solid was dissolved in a minimum of methanol (5 mL) and was precipitated upon addition of 40 mL diethyl ether. After 2 h, the precipitate was filtered, all solvents were reduced under pressure to afford a white hygroscopic solid as the compound (7). (0.192 g, 100%).

**^1^H NMR (300 MHz, D_2_O)**: δ 3.85 (d, *J* = 22.4 Hz, 6H), 3.58–3.29 (m, 13H), 3.06 (m, 9H), 2.54 (s, 1H). **^13^C NMR (75 MHz, D_2_O)**: δ 175.2, 171.8, 169.9, 163.2, 162.7, 122.0, 118.2, 114.3, 110.4, 79.7, 71.5, 56.4, 55.7, 53.6, 51.5, 50.6, 48.1, 28.8. **MS (ESI Positive)**: *m/z* 442.265 [M + H]^+^.

## Appendix C. Characterizations

### Appendix C.1. XPS

XPS characterizations are given for the Au bare gate (Table A1, Figure A3), for the Au gate modified with 1-azido-6-thiohexane (Table A2, Figure A4) and for the Au gate modified with the ionophore (Table A3, Figure A5).

**Table A1:** Characteristics of the XPS peaks, attribution and atomic percentage for a bare Au plate.

Name	Peak BE	FWHM eV	Area (P) CPS.eV	Atomic %
Au4f	84.24	0.77	1,622,127.82	46.89
C1s	284.42	1.31	24,840.75	16.77
C1s A	285.72	1.54	4611.59	3.12
C1s B	287.52	1.72	5281.57	3.57
N1s	399.25	0.4	2447.89	1.06
O1s	531.7	2.67	85,257.16	23.82
S2p Ox	167.76	0.28	1899.15	0.63

**Table A2:** Characteristics of the XPS peaks, attribution and atomic percentage for an Au surface modified with 1-azido-6-thiohexane (Product 3).

Name	Peak BE	FWHM eV	Area (P) CPS.eV	Atomic %
Au4f	84.18	0.77	1,753,345.31	50.44
C1s	284.76	1.39	29,150.61	19.59
C1s A	286.18	1.65	9128.36	6.14
C1s B	287.88	1.73	1898.79	1.28
N1s	399.19	1.72	2320.82	1
N1s A	400.99	1.9	9963.94	4.32
N1s B	404.3	1.43	2497.32	1.09
O1s	531.13	1.76	43,714.73	12.15
S2p	161.99	0.83	1882.86	0.62
S2p Ox	167.79	1.37	7713.57	2.57

As shown on Figure A4, the adsorption of the spacer is clearly visible on the N_1s_ and S_2p_ spectra. We found on the N_1s_ spectrum the particular signature of the 3 atoms of the azide function, with a band of strong binding energy at 404.3 eV corresponding to the central Nitrogen depleted in electrons (=N=) and a band of lower energy corresponding to the 2 satellite nitrogen atoms linked to the thiohexane chain (=N-), and to the most electronegative end of the azide (−N=) [33,34]. Analysis of the S_2P_ also confirms the immobilization of the spacer on the surface as demonstrated by the peak at 162.0 eV which corresponds to the signal of a thiol group bound to a gold surface by chemisorption, through an Au-S bond [35]. The absence of peak at 164 eV demonstrates that there is no simply adsorbed spacer. On the other hand, the presence of a peak at 167.8 eV shows that part of the thiols present is oxidized in the form of sulfonate or sulfinate [36,37]. The formation of these oxidized species is confirmed by the analysis of the O_1s_ spectrum on the unmodified Au and Au-Product 3, which presents a peak at 533 eV. Quantitative analysis of the atoms present also confirms the presence of a significant proportion of oxygen contamination.

The Table A3 shows the atomic composition of the electrode surface after coupling DO3A-alkyne to the 1-azido-6-thiohexane-modified electrode. It can be seen that the proportion of C_1s_ is considerably increased from 19.6% (for the 1-azido-6-thiohexane-modified electrode) to 39.5% for the DO3A-alkyne-modified electrode. Such an increase is explained by the presence of the 17 carbons of DO3A-alkyne added to the surface. The marked increase in O_1s_ between the 1-azido-6-thiohexane-modified electrode (12.1%) and the DO3A-alkyne-modified electrode (25.5%) confirms this hypothesis. The deconvolution of the C_1s_ spectrum shows 3 peaks identified as C_1s_, C_1sA_ and C_1sB_ (see Figure A5). The peak at 285.6–286.1 eV corresponding to the C-N bonds is present on the C_1s_ spectra of the 3 electrodes, but in totally different proportions [38]. This peak represents only 3% of the total on bare Au but increases to 6% for the 1-azido-6-thiohexane-modified electrode, to go up to 39% for the DO3A-alkyne-modified electrode. This variation is explained by the presence of C-N bonds at the level of the spacer but especially at the level of the DO3A-alkyne and the coupling arm. It is also interesting to note that the major peak at 284.4–284.7 eV corresponding to bonds of the C-C/C-H type [38] and present for bare Au and the 1-azido-6-thiohexane-modified electrode is no longer the major species on the DO3A-alkyne-modified electrode. The same for the peak at 287.4 eV corresponding to the presence of carbon linked to 2 single bonds such as O–C–O or a double bond with an oxygen in an amide function (NH_2_-C=O) or acetal or hemiacetal [39] on the different electrodes. However, this peak is present as trace on bare Au and 1-azido-6-thiohexane-modified electrode, whereas it represents 11% of the atoms present on the DO3A-alkyne-modified electrode, due to the grafted amide function. Finally, a peak at 289 eV corresponds to carbon making a double bond with an oxygen, characteristic of carboxylic acids or esters [36]. The peak at 162.1 eV for S_2p_ confirms that the spacer is still present on the surface. The N_1s_ spectrum shows that DO3A-alkyne is coupled to the azide spacer and not simply surface-adsorbed. Indeed, due to the conversion of the azide into a triazole function, the chemical environment of the nitrogen atoms involved evolves. This is the reason the high binding energy peak (404.3 eV) observed on the N_1s_ spectrum from the 1-azido-6-thiohexane-modified electrode disappears for the DO3A-alkyne-modified one, whereas the peak at 400.7 eV expands due to the formation of the triazole [33,34,40,41].

**Table A3:** Characteristics of the XPS peaks, attribution and atomic percentage for an Au surface modified with Product 7 after Click coupling.

Name	Peak BE	FWHM eV	Area (P) CPS.eV	Atomic %
Ag3d	368.05	0.78	78,864.56	1.83
Au4f	84.2	0.75	616,969.53	12.84
C1s	285.65	1.69	81,148.36	39.47
C1s A	287.44	1.74	24,231.7	11.8
C1s B	289.32	1.65	7701.99	3.75
N1s	400.7	1.91	10,018.3	3.14
O1s	533.76	1.86	126,405.99	25.46
S2p	162.09	0.9	3894.05	0.93
S2p Ox	169.19	1.14	2720.03	0.65

### Appendix C.2. FTIR

On the spectrum of the 1-azido-6-thiohexane-functionalized surface, the band at 2074 cm^−1^ corresponds to the azido group [42]; it is also visible on the spectrum of the DO3A-akyne-functionalized surface, but weaker, indicating that most but not all azido groups reacted upon coupling with the DO3A-alkyne. The red and green spectra can be superposed, except this azido group and the-C-N group at ca. 1050 cm^−1^. After coupling of the DO3A-alkyne, the broad band between 3000 and 3600 cm^−1^ increases, due to the hydroxyl group of the carboxylic acids of the DO3A molecule, as well as the band corresponding to the C=0 group at ca. 1714 cm^−1^ and the band at ca. 1450 cm^−1^ associated with the -CH_2_C=0 group of the same carboxylic group.

### Appendix C.3. Cyclic Voltammetry

The Fe(CN)_6_^3−^/Fe(CN)_6_^4−^ couple was used as redox probe to control the grafting of the 1-azido-6-thiohexane molecule as well as the coupling of the DO3A-alkyne onto it. The ∅1 mm Au wires (functionalized or not) were immersed in the Fe(CN)_6_^3−^/Fe(CN)_6_^4−^ solution and the potential was swept between −0.1 V and 0.7 V vs. Ag/AgCl. By comparison to the bare electrode, Figure A7 shows the evolution (lowering) of the electro-activity of the gold electrode upon the spacer grafting and the subsequent DO3A-alkyne coupling.
